# Neuropeptides in the Management of Alzheimer’s Disease: From Pathophysiology to Therapeutic Opportunities

**DOI:** 10.3390/ijms27073206

**Published:** 2026-04-01

**Authors:** Shraddha Tiwari, Shiv Kumar Yadav, Meenakshi Kumari, Thakur Dhakal, Nidhi Puranik

**Affiliations:** 1 Department of Life Sciences, Yeungnam University, Gyeongsan 38541, Republic of Korea; shraddha_tiwari@yu.ac.kr (S.T.);; 2 Department of Botany, Government Lal Bahadur Shastri P.G. College, Sironj 464228, India; 3 Department of Botany, Career Point University, Kota 324003, India

**Keywords:** neuropeptide, pituitary adenylate cyclase-activating polypeptide, neuropeptide Y, corticotropin-releasing factor, substance P, somatostatin, Alzheimer’s disease

## Abstract

Alzheimer’s disease (AD) is a progressive neurodegenerative disorder characterized by memory decline, cognitive impairment, and behavioral changes, ultimately leading to a loss of independence and reduced quality of life. Although understanding of the molecular basis of AD has advanced, effective disease-modifying therapies remain scarce. Neuropeptides are small protein-like signaling molecules that regulate diverse physiological processes, including mood, memory, and neuronal function. Growing evidence indicates that neuropeptides are promising therapeutic candidates for AD, particularly through modulation of neuroinflammation, synaptic plasticity, and amyloid-beta (Aβ) aggregation. Preclinical AD models show that neuroprotective neuropeptides, such as neuropeptide Y (NPY), vasoactive intestinal peptide (VIP), and pituitary adenylate cyclase-activating peptide (PACAP), exert neuroprotective effects, enhance memory, and attenuate cognitive decline. This review summarizes current research on neuropeptide-based therapies for AD, detailing their molecular mechanisms, therapeutic actions, and the barriers to their clinical translation. We specifically highlight neuropeptides whose clinical potential in AD remains comparatively underrecognized, discuss strategies for optimizing their delivery and overcoming pharmacokinetic limitations, and outline future perspectives for integrating neuropeptide-based interventions into AD therapy.

## 1. Introduction

Alzheimer’s disease (AD) is a progressive neurodegenerative disorder characterized by cognitive decline, memory impairment, and behavioral changes, ultimately leading to a loss of independence and reduced quality of life [1]. AD is the most common cause of dementia worldwide and represents a major public health challenge in aging populations [2]. The complex pathophysiology of AD is characterized by amyloid-beta (Aβ) plaque accumulation and neurofibrillary tangles formed by hyperphosphorylated tau, together with oxidative stress, neuroinflammation, and synaptic dysfunction [3,4,5,6]. These pathological changes cause widespread neuronal loss and disrupt synaptic transmission, particularly in memory- and learning-related regions such as the hippocampus and cortex [7]. Despite intensive efforts to develop AD therapies, cholinesterase inhibitors and N-methyl-D-aspartate (NMDA) receptor antagonists provide only modest symptomatic relief and do not halt disease progression. Consequently, research has shifted toward alternative therapeutic strategies that target core disease mechanisms, including neuropeptide-based approaches, because these molecules support neuronal homeostasis and modulate neurodegenerative pathways [8]. Neuropeptides act as neurotransmitters and neuromodulators in the central nervous system (CNS), contributing to neuroprotection, synaptic plasticity, and cognitive function, and they also regulate inflammatory processes [9]. Their long-lasting actions are mediated primarily through binding to G-protein-coupled receptors, which regulate numerous physiological processes. Several neuropeptides, including vasoactive intestinal peptide (VIP), pituitary adenylate cyclase-activating peptide (PACAP), neuropeptide Y (NPY), substance P (SP), and somatostatin (SST)—have attracted interest as potential AD therapies because they modulate amyloidogenic pathways, attenuate inflammatory responses, and improve synaptic function [10]. These neuropeptides exert neuroprotective effects by limiting cell death, controlling oxidative stress, and promoting neurogenesis, making them attractive therapeutic candidates. Aberrant neuropeptide signaling has been reported in AD brains, suggesting that restoring or enhancing these pathways may provide a novel disease-modifying strategy [10]. Accordingly, interest in neuropeptide-based treatments for AD is increasing as their diverse roles in brain regulation become better understood. However, clinical translation is still constrained by rapid peptide degradation, poor blood–brain barrier (BBB) penetration, and off-target adverse effects [11]. Advances in peptide engineering, nanotechnology-based delivery systems, and receptor-targeted strategies are beginning to address these limitations. Moreover, combining neuropeptide-based therapies with existing AD drugs may yield additive or synergistic effects that enhance disease modification [12,13].

Dementia is still a big public health problem around the world, with more than 55 million people living with it right now. About 60–70% of all cases of dementia are caused by AD. There are almost 10 million new cases diagnosed around the world every year, which is nearly one new case every three seconds. If the onset of dementia is not delayed, the number of cases around the world is expected to rise significantly, reaching 78 million by 2030 and 139 million by 2050. There were about 9.8 million cases of Alzheimer’s disease around the world in 2021. More than 60% of people with dementia live in low- and middle-income countries, which shows that there are big differences in healthcare access and resources. Women are more affected than men, with a higher age-standardized prevalence (770 per 100,000) compared to men (590 per 100,000). It was estimated that in 2025, about 7.2 million people in the United States who are 65 years old or older would have Alzheimer’s dementia. In 2022, AD was the sixth leading cause of death for people 65 and older. Between 2000 and 2022, the death rate more than doubled. It is important to note that one out of every three older Americans dies with Alzheimer’s disease or another type of dementia. As people get older, the risk of getting Alzheimer’s disease goes up a lot, which is one of the main reasons why the disease is becoming more common. China, India, and other countries in South Asia and the Western Pacific region are seeing the fastest growth in dementia cases. This is because of changes in the population and longer life expectancies in these areas [5].

In this review, we deliberately do not focus on extensively studied neurotrophic factors such as brain-derived neurotrophic factor (BDNF), nerve growth factor (NGF), and insulin-like growth factor-1 (IGF-1), which are already well represented in the AD literature. Although these molecules play critical roles in neuronal survival, synaptic plasticity, and neuroprotection, their mechanisms and therapeutic potential have been widely explored over recent decades. Instead, we highlight a comparatively underexplored set of neuropeptides—PACAP, VIP, substance P, neuropeptide Y, somatostatin, and CRF—that are increasingly recognized for their involvement in AD-related processes, including neuroinflammation, circadian rhythm regulation, sleep disturbances, stress responses, and neurodegeneration. By shifting the focus to these less conventional targets, we aim to underscore emerging evidence and encourage deeper investigation into alternative neuromodulatory pathways that may offer novel and complementary strategies for AD treatment.

This review provides an extensive analysis of the therapeutic potential of neuropeptides in AD by summarizing their mechanisms of action, reviewing ongoing preclinical and clinical studies, and outlining key barriers to drug development and clinical translation. By integrating these aspects, we aim to clarify how neuropeptide-based strategies could complement existing AD therapies and contribute to the development of more effective treatments for patients with this life-threatening disease.

## 2. Pathophysiology of Alzheimer’s Disease

The neurodegenerative condition AD manifests through a progressive pattern characterized by cognitive impairment and degeneration of brain cells [9]. AD develops as a result of amyloid-β (Aβ) plaque accumulation and neurofibrillary tangles formed from hyperphosphorylated tau protein which disrupt neuronal function and drive neurodegeneration [3,5,14]. The disease extends beyond protein aggregation, involving several interconnected pathological processes, including neuroinflammation, oxidative stress, synaptic dysfunction, and neurotransmitter imbalances [4,15]. These abnormal processes act synergistically to cause progressive deterioration of memory and other cognitive abilities. Current research highlights the critical importance of neuropeptides in maintaining brain homeostasis and identifies new avenues for modifying disease progression [16].

The main diagnostic feature of AD is the accumulation of Aβ peptides generated by two sequential processing steps of amyloid precursor protein (APP) via β-secretase (BACE1) and γ-secretase [17,18]. Pathological conditions lead to an imbalance between Aβ production and clearance, resulting in extracellular Aβ aggregates that evolve into plaques. These plaques disrupt synaptic communication and initiate toxic downstream processes [19]. Neuroinflammatory responses arise when plaques activate microglia and astrocytes, leading to further neuronal damage. In parallel with Aβ pathology, tau protein aggregates into neurofibrillary tangles that disrupt microtubules, block axonal transport, and ultimately cause neuronal death [16]. Tau pathology correlates closely with synaptic failure and cognitive decline, and accumulating evidence indicates that tau spreads between brain regions in a prion-like manner.

Neuroinflammation is a key contributor to AD pathogenesis. Activation of microglia and astrocytes in response to Aβ accumulation induces the release of pro-inflammatory cytokines, including interleukin-1β (IL-1β), tumor necrosis factor-alpha (TNF-α), and interleukin-6 (IL-6) [20,21,22]. While acute neuroinflammation can have protective effects, chronic inflammation exacerbates neuronal and synaptic damage. Oxidative stress significantly accelerates AD development because reactive oxygen species (ROS) damage lipids, proteins, and DNA [23,24,25,26].

**Figure 1:**
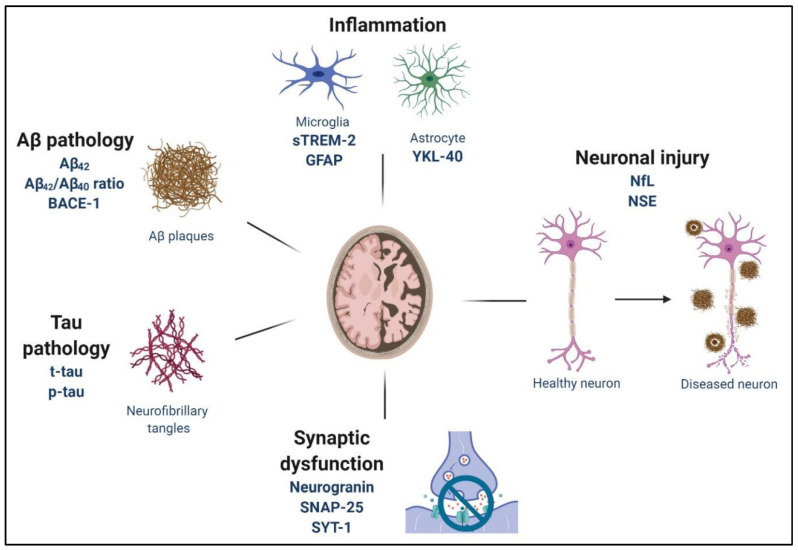
The figure summarizes major pathological pathways in Alzheimer’s disease and their related biomarkers. Amyloid-β alterations (Aβ_42_, Aβ_42_/Aβ_40_ ratio, BACE-1) contribute to plaque formation, while increased total and phosphorylated tau promote neurofibrillary tangles. Neuroinflammation involves activated microglia and astrocytes, indicated by sTREM-2, GFAP, and YKL-40. Synaptic impairment is reflected by neurogranin, SNAP-25, and SYT-1, and neuronal damage is marked by elevated NfL and NSE. These interconnected processes collectively drive neurodegeneration and cognitive decline in AD. The figure is adapted from [29].

AD pathology is further characterized by synaptic dysfunction and chemical imbalances at synapses. Cognitive impairment arises from a loss of synaptic integrity, particularly in the hippocampus and cortex. The cholinergic system, which supports learning and memory, is severely affected because basal forebrain cholinergic neurons degenerate and produce less acetylcholine. In addition, AD involves dysregulation of the glutamatergic system, leading to excitotoxic neuronal injury and death [27,28]. Degeneration of serotonergic, dopaminergic, and GABAergic systems further contributes to cognitive deficits and behavioral disturbances. The major pathological mechanisms occurring in AD are summarized in Figure 1.

The pathological changes in brain tissue are regulated by neuropeptides, which help maintain brain homeostasis and control key processes related to AD development [30,31]. Vasoactive intestinal peptide (VIP), pituitary adenylate cyclase-activating polypeptide (PACAP), neuropeptide Y (NPY), and somatostatin (SST) have been reported to modulate synaptic plasticity, limit neuroinflammation, and exert protective effects against oxidative stress. VIP and PACAP also protect neurons from the neurotoxic effects of Aβ by promoting neuronal survival and preserving synaptic function and integrity [32,33]. In addition to its role in stress regulation, NPY helps preserve brain function by maintaining the excitatory–inhibitory balance in neuronal circuits [34,35]. Similarly, SST is important for modulating memory-related processes and Aβ metabolism, in part by enhancing Aβ clearance [36,37,38].

Dysregulation of neuropeptide signaling in the AD brain promotes the progression of neurodegenerative processes, leading to synaptic loss and impaired neuronal communication. Given their diverse neuroprotective actions, neuropeptides represent promising therapeutic targets to modify disease course and restore cognitive function in patients with AD [39]. Understanding the multifactorial nature of AD, therefore, requires detailed insight into the role of neuropeptides and their differential effects on Aβ and tau pathology, neuroinflammation, oxidative stress, and synaptic dysfunction. Although most current therapeutic strategies aimed at preserving brain homeostasis focus on targeting Aβ and tau, recent studies suggest that correcting neuropeptide dysregulation offers a novel approach to counteracting brain dysfunction and preventing neurodegenerative changes. Elucidating how neuropeptides influence AD pathology will facilitate the development of neuropeptide-based therapeutics that could slow or even prevent disease progression.

## 3. Neuropeptides and Their Roles in AD

Neuropeptides hold strong potential for clinical application, offering advantages such as fewer side effects and easier chemical modification compared with proteins or monoclonal antibodies. However, their use is often limited by low metabolic stability and a short duration of action.

### 3.1. Vasoactive Peptide in AD

Vasoactive peptides are a diverse group of molecules that regulate vascular tone and act as peripheral neurotransmitters, particularly in pain modulation. Based on their effects, they are classified as vasodilators or vasoconstrictors. Beyond their vascular roles, recent studies have highlighted their central nervous system functions, including acting as growth factors. Disruptions in vasoactive peptide signaling may contribute to neurodegenerative diseases such as AD by altering neuronal stress responses, survival pathways, and synaptic plasticity [40].

The 28-amino-acid neuropeptide VIP is widely distributed in the central nervous system and participates in neuronal survival, neuroprotection, anti-inflammatory responses, and immune modulation [41,42]. VIP is highly expressed in the cerebral cortex, hippocampus, and hypothalamus, brain regions that undergo substantial degeneration in AD. The effects of this neuropeptide are mediated via binding to G protein-coupled receptors (GPCRs)—VPAC1, VPAC2, and PAC1—which have been identified in multiple tissues, including the brain [10], and regulate diverse cellular processes such as neuronal differentiation, synaptic plasticity, and neuroimmune signaling [42,43]. VIP is also widely expressed in peripheral tissues and is involved in numerous physiological functions. Consequently, VIP has attracted considerable interest because of its broad biological actions and potential as a therapeutic agent for neurodegenerative disorders, including AD.

**Figure 2:**
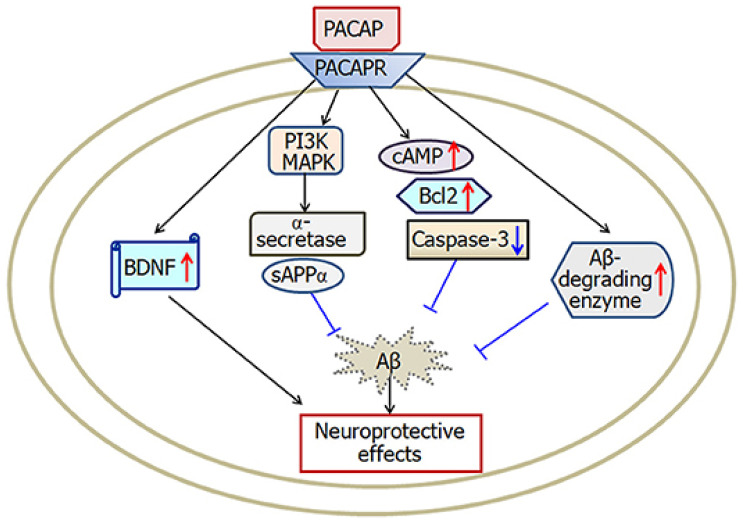
PACAP binds to PAC1 receptors, activating PI3K/MAPK and cAMP pathways to increase sAPPα, BDNF, and Bcl-2, inhibit caspase-3, and enhance Aβ degradation, collectively reducing apoptosis and Aβ accumulation while promoting neuronal survival and neuroprotection [72].

VIP acts as an important protective factor against Aβ pathology in AD. Experimental studies have shown that VIP reduces Aβ-induced neurotoxicity by enhancing Aβ clearance and limiting Aβ aggregation (Figure 2) [33,43]. This effect is mediated in part by upregulation of insulin-degrading enzyme (IDE) and neprilysin (NEP), two key proteases responsible for Aβ degradation. VIP also increases levels of brain-derived neurotrophic factor (BDNF), a critical mediator of neuronal survival and synaptic plasticity that is typically reduced in patients with AD. In addition, VIP preserves synaptic connectivity and prevents Aβ-induced neurodegeneration that underlies cognitive impairment.

Beyond its direct effects on Aβ metabolism, VIP is a potent modulator of neuroinflammation, which is central to AD progression. Toxic Aβ deposits induce chronic activation of microglia and astrocytes, which secrete pro-inflammatory cytokines such as TNF-α, IL-1β, and IL-6, thereby damaging neurons and contributing to cognitive decline. VIP exerts strong anti-inflammatory actions by modulating microglial and astrocytic activation and reducing the production of pro-inflammatory cytokines. It also promotes the release of anti-inflammatory cytokines such as IL-10 and fosters a pro-regulatory immune environment that limits excessive neuroinflammation. Furthermore, VIP inhibits the nuclear factor kappa B (NF-κB) signaling pathway, a key transcriptional regulator of neuroinflammatory responses, thereby attenuating the activation of inflammatory mediators that drive neurodegeneration in AD. VIP additionally exhibits cerebrovascular protective effects, which are particularly relevant because cerebrovascular dysfunction is a prominent feature of AD [44].

AD-associated vascular dysfunction reduces cerebral blood flow, compromises oxygen and nutrient delivery to neurons, and increases blood–brain barrier (BBB) permeability, thereby aggravating disease pathology. VIP has vasodilatory properties and improves cerebral perfusion by increasing nitric oxide (NO) availability and enhancing endothelial function. These effects improve oxygenation and nutrient supply to brain tissue, reduce neuronal stress, and help prevent further cognitive decline [45,46]. VIP also contributes to maintaining BBB integrity and limiting peripheral immune cell infiltration, which otherwise amplifies neuroinflammation in AD [41,47].

AD is characterized by severe degeneration of cholinergic neurons in the basal forebrain and a resulting cholinergic deficit. VIP stimulates choline acetyltransferase synthesis, increasing expression of choline acetyltransferase (ChAT), promoting acetylcholine production, and improving cognitive functions related to learning and memory [48,49]. Moreover, VIP interacts with the glutamatergic system by modulating N-methyl-D-aspartate (NMDA) receptor activity, thereby limiting excitotoxicity, a major contributor to neuronal loss in AD [50]. By restoring a more balanced neurotransmitter profile, VIP may help ameliorate cognitive deficits.

Despite these promising properties, translation of VIP into effective clinical therapy for AD faces several challenges. The primary limitation is the short half-life of VIP, which is rapidly degraded by peptidases, resulting in low bioavailability in the central nervous system [51]. In addition, VIP exhibits poor BBB penetration, making systemic administration inefficient for targeting neuronal pathology in AD [52]. To overcome these obstacles, researchers are developing novel delivery strategies, including VIP analogs with increased metabolic stability, nanoparticle-based formulations, and intranasal administration to enhance central nervous system penetration [53]. VIP receptor agonists are also being explored as an alternative means to harness VIP-like therapeutic effects while mitigating the issue of peptide degradation [54]. Overall, VIP shows considerable promise for the treatment of inflammatory, neurodegenerative, and cancer-related conditions, but translating its preclinical efficacy into clinical practice remains challenging, as is common for many neuropeptides [55]. VIP is a promising neuropeptide candidate for therapeutic targeting of AD because it exerts multiple effects, including neuroprotection, anti-inflammatory activity, synaptic protection, cerebrovascular regulation, and neurotransmitter modulation. Alongside efforts to reduce Aβ toxicity, modulate neuroinflammation, improve cholinergic function, and promote vascular integrity, VIP has the potential to slow or even halt disease progression [56,57].

### 3.2. Pituitary Adenylate Cyclase-Activating Polypeptide (PACAP)

PACAP is a conserved neuropeptide that influences neuronal activity and gene expression via Gs/Gq-coupled receptors. It plays vital roles in stress regulation, emotional processing, neuroprotection, and cognition, particularly through its action in the hypothalamus, limbic system, and memory-related brain regions. Elevated PACAP levels and altered PAC1 receptor function have been linked to abnormal fear responses and maladaptive memory in PTSD, highlighting its role in integrating stress and traumatic memory. Although its involvement in stress has been well studied, PACAP’s role in memory and learning—especially via PAC1 signaling—warrants further research. Emerging findings also suggest sex-specific differences in PACAP signaling, pointing to critical areas for future investigation in both adaptive and pathological fear learning [58].

Recent in vitro and in vivo studies have highlighted the strong neuroprotective properties of PACAP. Notably, PACAP appears capable of crossing the BBB in biologically relevant amounts, making it a promising therapeutic candidate for neurodegenerative disorders. Research using models of AD and PD shows that PACAP administration can reduce pathological changes and improve clinical outcomes, supporting its potential as a novel treatment strategy for these conditions [59].

PACAP is a member of the VIP/secretin/glucagon family of neuropeptides and exhibits powerful neuroprotective, neurogenic, and anti-inflammatory properties [60]. Two biologically active forms of PACAP have been identified, PACAP-27 and PACAP-38, of which PACAP-38 is the predominant form in the CNS. PACAP exerts its effects by binding to three GPCRs—PAC1, VPAC1, and VPAC2—among which PAC1 has the highest affinity [61]. PACAP is widely expressed in brain regions involved in cognitive function, such as the hippocampus, cortex, and hypothalamus, and is implicated in neurogenesis, synaptic plasticity, and Aβ metabolism [62]. A major contribution of PACAP to potential AD therapy is its capacity to stimulate neurogenesis, the formation of new neurons from neural stem cells [63]. Neurogenesis is required for the maintenance of cognitive function, memory formation, and synaptic plasticity, all of which are also impaired in AD. PACAP promotes neural progenitor cell proliferation and differentiation in the hippocampus, a brain region that is severely affected by AD-related degeneration. PACAP supplementation has been shown to promote the expression of neurotrophic factors, notably BDNF, nerve growth factor (NGF), and glial cell line-derived neurotrophic factor (GDNF), thereby supporting neuronal survival, synaptic connectivity, and repair mechanisms [64]. PACAP counteracts the progressive neuronal loss associated with AD and, by promoting neurogenesis, may help reverse aspects of memory deficits in affected individuals [65]. PACAP is not only neurogenic but also involved in Aβ clearance, which is essential for slowing AD progression. Accumulation of Aβ peptides, resulting from an imbalance between their production and clearance, contributes to the formation of toxic plaques that disrupt synaptic function and trigger neurodegenerative cascades [65]. It has been shown that PACAP potentiates the degradation and clearance of Aβ peptides through several pathways. It upregulates the expression of enzymes that break down extracellular Aβ aggregates—for example, the Aβ-degrading enzymes neprilysin and insulin-degrading enzyme [66]. In addition, PACAP regulates autophagy-related pathways, leading to the degradation of misfolded proteins inside cells. PACAP promotes Aβ clearance, thereby lowering plaque burden and alleviating the associated neurotoxicity and loss of synaptic function in AD brains [66]. In addition to its direct effects on neurogenesis and Aβ metabolism, PACAP is strongly neuroprotective, in part through its anti-inflammatory and antioxidant mechanisms. Activation of microglia and astrocytes causes chronic neuroinflammation that plays a major role in neurodegeneration in AD [67]. When activated, microglia release pro-inflammatory cytokines such as TNF-α, IL-1β, and IL-6, thereby exacerbating synaptic dysfunction and neuronal apoptosis [67]. In this context, PACAP has been shown to inhibit microglial activation and decrease the production of these pro-inflammatory cytokines, while increasing the production of anti-inflammatory mediators such as IL-10 [68]. This shift toward an anti-inflammatory environment protects neurons from toxicity due to chronic inflammation. PACAP also serves as a potent antioxidant by decreasing the formation of ROS and increasing the activity of antioxidant enzymes, including superoxide dismutase (SOD) and glutathione peroxidase [69]. PACAP also plays an essential role in the pathophysiology of AD by regulating synaptic plasticity and neurotransmitter balance [70]. PACAP modulates the activity of the glutamatergic and cholinergic systems, which are severely impaired in AD. PACAP modulates NMDA receptor activity and prevents excitotoxicity that results from glutamate overstimulation of neurons [71]. PACAP also enhances cholinergic function by elevating acetylcholine synthesis and release, and may therefore compensate for the cholinergic deficits that occur in AD. These effects appear to help preserve cognitive function and delay disease progression [71]. A schematic representation of the mechanisms underlying PACAP-mediated neuroprotective effects in AD is shown in Figure 2.

Despite its immense therapeutic potential, the clinical application of PACAP faces several challenges. Like other neuropeptides, PACAP has a short half-life because rapid enzymatic degradation limits its bioavailability [73]. In addition, PACAP exhibits poor permeability across the BBB, rendering systemic administration largely ineffective [74]. To overcome these barriers, researchers are investigating novel drug delivery strategies, including PACAP analogs with improved stability, nanoparticle-based carriers, and intranasal administration to deliver PACAP directly to the CNS [75]. Furthermore, the neuroprotective potential of PACAP has generated interest in receptor agonists as an alternative therapeutic approach that avoids peptide degradation.

In summary, PACAP is a highly promising neuropeptide therapeutic candidate for AD because it contributes to neurogenesis, Aβ clearance, synaptic plasticity, and neuroprotection. PACAP has the potential to inhibit AD pathology by stimulating neural stem cell proliferation, enhancing Aβ degradation, modulating neuroinflammation, and preventing oxidative stress. Recent advances have led to the development of synthetic PACAP analogs with improved pharmacological profiles. These findings highlight PACAP receptor activation as a promising therapeutic strategy for cognitive disorders, including FXS [76].

### 3.3. Mechanism of PACAP-Mediated Misfolded Protein Degradation

**Figure 3:**
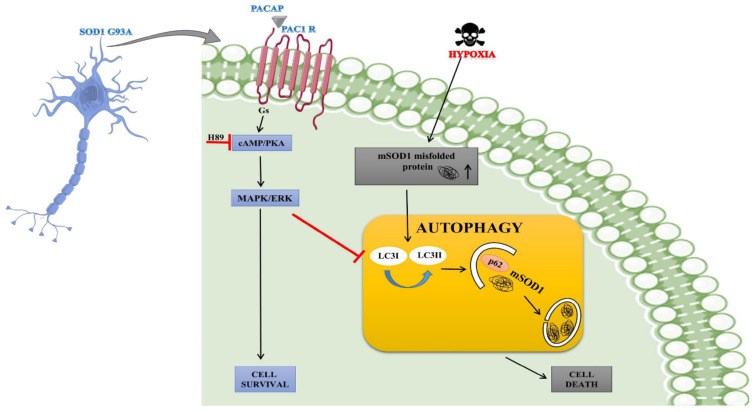
PACAP binds to the PAC1 receptor and activates cAMP-dependent signaling, including the MAPK/ERK pathway, which regulates autophagy under stress conditions. This controlled autophagy promotes the clearance of misfolded proteins and enhances cell survival in neurodegenerative models [79].

PACAP helps by increasing the power of cell cleanness and activating the natural cleaning process, which is known as autophagy. Autophagy serves as a dustbin and recycling system for cells. It removes damaged proteins and waste material from cells [77]. The interplay between PACAP/PAC1 receptor signaling, mutant SOD1 (SOD1-G93A) toxicity, hypoxia, and autophagy has been demonstrated in a neuronal model relevant to amyotrophic lateral sclerosis (ALS). In SOD1-G93A motor neurons, hypoxic stress promotes the accumulation of misfolded mutant SOD1 (mSOD1) protein, which contributes to cellular toxicity. In response to protein misfolding, the autophagic pathway is activated as a protective mechanism. During autophagy, LC3-I is converted to LC3-II, facilitating autophagosome formation, while the adaptor protein p62 binds to misfolded mSOD1 and directs it toward autophagic degradation. Controlled autophagy helps clear toxic aggregates and supports cell survival; however, excessive or dysregulated autophagy can contribute to cell death. Simultaneously, PACAP (pituitary adenylate cyclase-activating polypeptide) binds to its Gs-coupled PAC1 receptor on the neuronal membrane, activating the cAMP/PKA signaling cascade, which subsequently stimulates the MAPK/ERK pathway. Activation of ERK promotes neuronal survival (Figure 3). The diagram below suggests that ERK signaling negatively regulates excessive autophagy, thereby preventing autophagy-mediated cell death [78].

### 3.4. Substance P (SP)—Neuroinflammation and Glial Activation in AD

SP belongs to the tachykinin family of 11-amino-acid neuropeptides and plays important roles in pain signaling, stress responses, and neuroinflammation [76]. It exerts its biological effects by interacting with the neurokinin-1 receptor (NK1R), which is highly expressed in the CNS and peripheral tissues. SP has been implicated in the modulation of neuroinflammatory responses and glial activation, both of which are important for AD progression [78].

In AD, SP levels in the brain and cerebrospinal fluid are altered. SP and NK1R are closely linked to Toll-like receptor (TLR) signaling, forming a bidirectional regulatory loop that contributes to AD pathophysiology. SP has been shown to influence the non-amyloidogenic pathway and modulate voltage-gated potassium channel currents, underscoring its neuroprotective role. Emerging evidence supports a strong connection between SP, NK1R, and TLR pathways in the context of AD [80].

SP appears to exert neuroprotective effects in both AD and PD, as suggested by its reduced levels in affected brain regions. In AD, SP helps counteract Aβ toxicity by modulating voltage-gated potassium (Kv) channels, supporting its anti-amyloidogenic role. In PD, SP is implicated in both motor and nonmotor symptoms, such as olfactory and gastrointestinal dysfunctions. It colocalizes with α-synuclein aggregates in key regions, including the olfactory bulb and vagus nerve. These findings suggest that SP may serve as both a prognostic biomarker and a potential therapeutic target in AD and PD [60].

Inflammatory responses from CNS cells are crucial contributors to the onset and progression of various neuroinflammatory and neurodegenerative disorders, including AD, PD, MS, and encephalitis. SP, a neuropeptide highly expressed in the CNS, and its receptor, NK1R, which is found on neurons, microglia, and astrocytes, have emerged as key modulators of these responses. While SP is known for its roles in pain perception and gut motility, it also amplifies inflammation in both peripheral and central tissues. Recent studies show that SP–NK1R signaling enhances immune activity in glial cells, potentially exacerbating neuroinflammation in infectious and sterile CNS diseases. These findings highlight the therapeutic potential of repurposing NK1R antagonists—currently used for nausea and anxiety—as adjunct treatments to reduce CNS inflammation in such conditions [61]. The hippocampal CA2 region, located between CA1 and CA3, has historically been understudied with respect to its role in memory. This area receives input from supramammillary axons rich in SP, a neuropeptide that acts as both a neurotransmitter and a neuromodulator. Research shows that SP enhances synaptic strength at CA2 synapses through mechanisms dependent on NMDA receptors, protein synthesis, and kinases such as CaMKIV and PKMζ. Notably, SP converts short-term potentiation in the entorhinal cortex–CA2 pathway into long-term potentiation, supporting synaptic tagging and capture—an associative process essential for memory consolidation.

The hippocampal CA2 region, which is crucial for social behavior, receives input from SP-expressing neurons of the supramammillary (SuM) nucleus. SP modulates pain and central synaptic transmission by inducing a gradual, NMDA receptor- and protein synthesis-dependent potentiation at both entorhinal cortex (EC)-CA2 and normally LTP-resistant Schaffer collateral (SC)-CA2 synapses. SP also converts short-term potentiation at EC-CA2 synapses into long-term potentiation (LTP), supporting the synaptic tagging and capture model. This potentiation occurs independently of GABAergic inhibition and relies on the kinases CaMKIV and PKMζ. Thus, SuM-derived SP primes CA2 synapses for durable synaptic plasticity and associative integration [81].

There is increasing evidence that SP contributes to AD pathology primarily by modulating neuroinflammation. A hallmark of AD is chronic neuroinflammation, characterized by persistent microglial and astrocytic activation in response to Aβ deposition [62]. SP promotes the release of pro-inflammatory cytokines, such as TNF-α, IL-1β, and interleukin-6 (IL-6), from glial cells. In turn, these cytokines exacerbate synaptic dysfunction, neuronal apoptosis, and BBB disruption [62]. Beyond cytokine release, SP also induces microglial and astrocytic activation, leading to increased production of ROS and nitric oxide (NO), which contribute to oxidative stress and neuronal damage. Because NK1R is the main receptor for SP, pharmacological blockade of NK1R in microglia can attenuate microglial activation and decrease neuroinflammation in neurodegenerative models. Therefore, targeting SP-NK1R signaling may represent a potential therapeutic strategy to counter neuroinflammation in AD [63]. Although SP has mainly been known to exert pro-inflammatory effects, some studies have indicated that it may also act in a dual manner in AD [64]. Under specific conditions, SP can promote neuroprotection by modulating synaptic plasticity and enhancing neuronal survival. The intricate balance between neuroprotection and neuroinflammation mediated by SP during AD progression underscores the need for further research to define how SP can be harnessed therapeutically [65].

### 3.5. Neuropeptide Y

NPY is widely distributed in the CNS, especially in the hippocampus, cortex, and amygdala, brain regions that mediate learning, memory, and emotional regulation [66]. NPY is a 36-amino-acid neuropeptide that binds to G protein-coupled receptors (Y1, Y2, Y4, and Y5), among which Y1 and Y2 are the most relevant in AD pathology [67]. NPY participates in multiple physiological functions, including appetite regulation, stress responses, and synaptic plasticity; therefore, it is an essential modulator of processes implicated in AD. NPY plays an important role in promoting synaptic plasticity underlying learning and memory formation, which is one of its main contributions to potential AD therapy [68]. In AD, there is a progressive loss of synaptic integrity, resulting in cognitive decline. It has been demonstrated that NPY increases dendritic spine density, modulates excitatory and inhibitory neurotransmission, and enhances hippocampal LTP.

Apart from its role in synaptic function, NPY is a well-known modulator of the stress response [69]. Chronic stress is a risk factor for AD because it leads to hippocampal atrophy, neuroinflammation, and Aβ accumulation. Conversely, NPY acts as an anxiolytic and neuroprotective agent by reducing the release of CRF and thereby decreasing stress-induced cortisol levels [70]. This stress-modulating role of NPY could counteract the detrimental effects of chronic stress on the brain and slow AD progression. NPY-based therapies for AD have been investigated experimentally. Administration of exogenous NPY in such models improves cognitive performance, decreases neuroinflammation, and prevents Aβ toxicity. Targeting NPY signaling pathways may therefore present a novel therapeutic approach to treating AD, particularly in individuals who are highly susceptible to stress.

### 3.6. Corticotropin-Releasing Factor (CRF)

CRF and its related peptides, urocortins 1–3 (UCN1–UCN3), regulate stress responses by activating two class B GPCRs, CRF1R and CRF2R. Cryo-electron microscopy has resolved the structures of UCN1-bound CRF1R and CRF2R complexes with stimulatory G proteins. In both receptors, UCN1 forms a single helix that inserts its N-terminus into the transmembrane domain. While peptide-binding residues differ from those of other class B GPCRs, key residues for receptor activation and G protein coupling are conserved. The structures also reveal cholesterol molecules interacting with the receptor helices. These findings clarify ligand specificity, unify the mechanism of class B GPCR activation and G protein engagement, and offer insights into membrane protein–lipid interactions [71]. CRF is a key regulator of the hypothalamic–pituitary–adrenal (HPA) axis and plays a central role in the body’s stress response. Dysregulation of CRF signaling has been linked to depression. A review highlighted the involvement of CRF in neuroimmune pathways contributing to depression and explored the potential of CHM and other treatments to modulate this system. Understanding the interplay between CRF and immune responses may deepen insights into depression’s underlying mechanisms and guide the development of new therapeutic approaches [73].

CRF is a crucial neuropeptide regulating physiological, endocrine, and behavioral stress responses. Dysregulation of the CRF system is linked to affective disorders such as PTSD, depression, and anxiety, which are also associated with cognitive impairments for which effective treatments are limited. One review emphasizes CRF’s significant influence on higher cognitive functions through its actions in the prefrontal cortex and subcortical monoaminergic and cholinergic pathways. Key future research directions include exploring sex differences in CRF’s cognitive effects and assessing CRF as a potential pharmacological target to alleviate cognitive deficits. Advancing this knowledge may improve therapeutic strategies for neuropsychiatric conditions involving cognitive dysfunction [74].

CRF and its related peptides, urocortins 1–3, along with their receptors corticotropin-releasing factor receptors 1 and 2 (CRFR1 and CRFR2), form a key neuropeptidergic system that orchestrates the physiological stress response. This system integrates autonomic, neuroendocrine, and behavioral adaptations that are critical for coping with stress. This review provides a comprehensive update on the molecular biology, gene regulation, structural features, and signaling mechanisms of CRF peptides and receptors. It also highlights recent advances using precise genetic tools to elucidate their specific roles in stress regulation. Current controversies and knowledge gaps are discussed, offering directions for future research and potential therapeutic targets [75].

**Figure 4:**
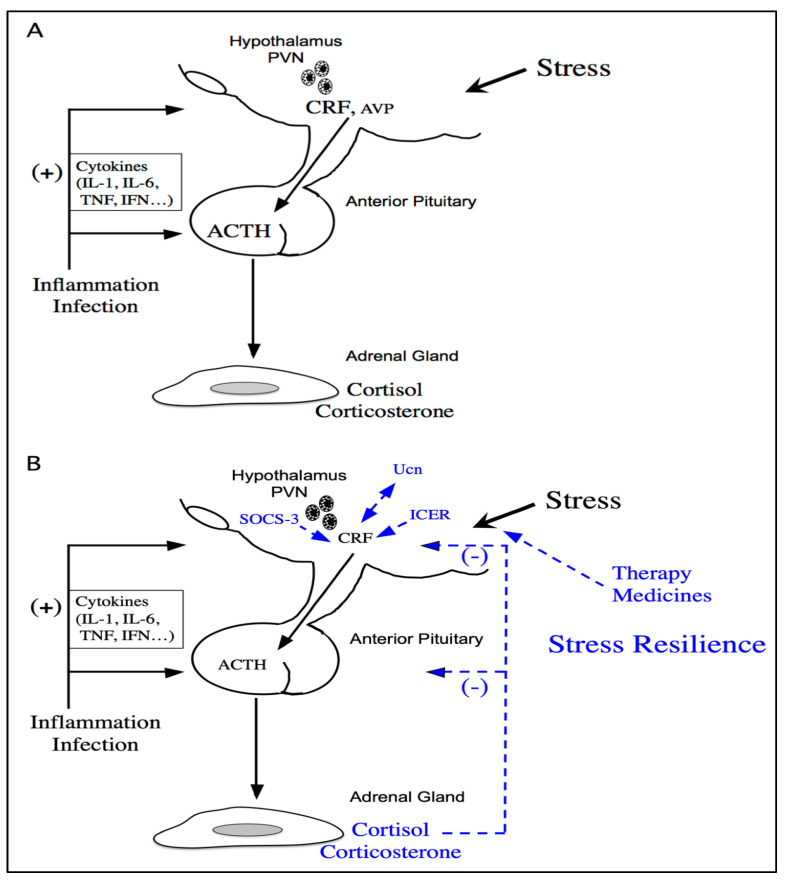
Hypothalamic-pituitary-adrenal axis dynamics stress response and recovery. (**A**) Activation: Hypothalamic CRF and cytokines drive ACTH and adrenal glucocorticoid secretion. (**B**) Resilience: ICER and SOCS-3 provide negative feedback on CRF synthesis, while the urocortin-CRF2 pathway mediates adaptive coping mechanisms. Modulation of these regulatory elements offers therapeutic potential for stress-related pathologies [82].

CRF in the hypothalamus plays a pivotal role in regulating the stress response by stimulating adrenocorticotropic hormone (ACTH) release, which in turn triggers glucocorticoid secretion from the adrenal glands. Glucocorticoids promote stress coping, resilience, and homeostasis and inhibit the HPA axis through negative feedback (Figure 4). This repression involves glucocorticoid response elements and regulators such as the inducible cAMP-early repressor and suppressor of cytokine signaling-3. CRF receptor type 1 primarily mediates stress-related effects, including depression and seizures, whereas CRF receptor type 2 facilitates stress coping, including anxiolytic effects. Additionally, the immunophilins FKBP4 and FKBP5 modulate glucocorticoid signaling, contributing to individual differences in stress resilience. Together, these factors coordinate the complex processes underlying stress adaptation [76].

CRF1 is broadly expressed in brain regions that regulate both immediate and long-term responses to social and environmental stressors, which are key contributors to major depressive disorder (MDD) development. A review from the 2014 International Behavioral Neuroscience Society symposium consolidates the current understanding of CRF physiology and its impact on MDD symptoms, drawing on findings from multiple laboratories represented at the symposium. The accumulated evidence underscores substantial advances in MDD research and points toward new directions for diagnosis and treatment [77].

Stress is a fundamental part of daily life, with the neuropeptide CRH playing a central role in coordinating neuroendocrine, autonomic, and behavioral responses. Produced primarily by neurons in the hypothalamic PVN, CRH activates the HPA axis to initiate stress responses. Outside the hypothalamus, CRH in other brain regions acts as a neuromodulator, shaping communication between hormonal and behavioral stress responses. CRH-expressing neurons project to various brain areas, interacting with CRHR1 and CRHR2 receptors and thereby recruiting multiple signaling systems that mediate diverse stress-related responses. The impact of stress on brain function ranges from promoting adaptive behavior and survival to increasing the risk of mental health disorders. Dysregulation of the corticotropin-releasing hormone (CRH) system is closely linked to conditions including depression, anxiety, addiction, neuroendocrine imbalances, inflammation, and neurodegenerative diseases such as AD. Current research has targeted CRHR1 receptors with antagonists as potential antidepressant treatments. Clarifying how the CRH system and its receptors contribute to detrimental effects on hormone-regulated systems, and how these effects can be prevented, is a key focus in physiological genetics. A review by Sukhareva highlights CRH’s role in stress regulation and examines how extrahypothalamic CRH contributes to the pathophysiology of mental disorders and to therapeutic approaches [78].

Recent research indicates that individuals with post-traumatic stress disorder (PTSD) are at higher risk of developing dementia, particularly AD, later in life. One relevant study examined whether PTSD symptoms exacerbate AD progression or whether both disorders share underlying vulnerabilities. Using mouse models, including wild-type animals and genetically modified AD models, researchers found that mice carrying familial AD mutations became more sensitive to trauma-induced PTSD-like behaviors and stress hormone changes. PTSD-like trauma increased cerebrospinal fluid levels of Aβ, which accelerated AD pathology. These effects depended on CRFR1 signaling and an intact HPA axis. Additionally, Aβ itself increased the excitability of CRF neurons, linking AD pathology to dysregulation of stress circuits. Reducing Aβ levels during trauma exposure alleviated PTSD-like symptoms. Together, these findings suggest that PTSD-like stress can promote AD progression by disrupting CRF signaling, thereby intensifying PTSD symptoms and raising the risk of dementia [80].

CRF, a central hormone and neuropeptide in stress responses, is dysregulated in AD pathology. Despite this, the exact roles of CRF and its binding protein (CRF-BP) in AD are not well understood. Stress-induced modulation of the CRF system affects AD pathogenesis and highlights the potential of targeting CRF-BP as a novel therapeutic strategy for AD [77]. A recent study revealed a sex difference in CRF1 receptor signaling in females, CRF1 preferentially couples to the G protein Gs, whereas in males, it couples more strongly to β-arrestin-2. Using phosphoproteomic analysis in CRF-overexpressing mice, researchers found that this sex-biased receptor coupling leads to distinct brain phosphoprotein profiles. In females, phosphoproteins linked to AD pathways were increased, including tau phosphorylation and β-secretase phosphorylation—key factors in AD pathology. Female mice exhibited greater amyloid β plaque formation and more pronounced cognitive decline than males. These findings suggest that excess CRF triggers sex-specific cellular processes via CRF1, potentially explaining the greater female susceptibility to AD through promotion of AD-related signaling pathways [83].

Another study evaluated the effects of R121919, a selective antagonist of CRFR1, on AD progression by administering it to transgenic AD mice starting at 30 days of age and continuing for 5 months. Treatment with R121919 significantly prevented cognitive decline in female mice. In both male and female mice, it reduced cellular and synaptic deficits and decreased amyloid beta and C-terminal fragment-β accumulation, without causing any toxicity or tolerability issues. These findings suggest that CRFR1 antagonism is a promising disease-modifying approach for AD and support further investigation in early-phase human clinical trials [84].

Reports from AD biomarker studies highlight a strong connection between oxidative stress and AD neuropathology, with the antioxidant glutathione (GSH) playing a critical role in defending against ROS and maintaining redox balance. Previous research has shown that oxidative stress, marked by increased S-glutathionylated proteins (Pr-SSG), precedes overt AD pathology. This oxidative stress may also disrupt the HPA axis, activating inflammatory pathways and elevating CRF production. To test whether reducing central CRF signaling can mitigate oxidative damage, the selective CRFR1 antagonist R121919 was administered to AD-transgenic mice from 30 days of age for 150 days. Treatment with R121919 significantly decreased Pr-SSG levels and enhanced glutathione peroxidase activity, indicating that CRFR1 blockade may serve as a preventive strategy to reduce oxidative stress in AD [85].

A recent study examined alterations in pre- and postsynaptic markers of CRF across several neurodegenerative disorders, including AD, PD, progressive supranuclear palsy (PSP), and HD [72,83].

### 3.7. Somatostatin and Somatostatinergic Interneurons in AD

Research on SST in AD began gaining significant attention in the 1980s, revealing consistent colocalization of SST with Aβ and notable losses of SST and SST-expressing cells (SST-INs). While early studies focused on the neuroendocrine and hypothalamic roles of SST, understanding of the GABAergic interneurons producing SST in cortical and hippocampal areas remained limited. Over the past four decades, interest has surged as SST-INs have emerged both as promising therapeutic targets and as key contributors to AD pathogenesis. This perspective updates a molecular model that positions SST-IN hyperactivity at the very earliest stage (“stage 0”) of neurodegeneration and proteinopathy in AD, offering a parsimonious explanation—aligned with Occam’s razor—for the differential regional patterns observed, notably the paradoxical accumulation of phosphorylated tau in the hippocampus versus Aβ in the cortex [36].

The neuropeptide SST has important functions in neurotransmission, neuroprotection, and Aβ metabolism. These effects involve five somatostatin receptor subtypes (SSTR1–SSTR5), which are widely expressed in the cortex and hippocampus [38]. SST is best known for its inhibitory effects on the release of neurotransmitters (e.g., GABA and glutamate), through which it helps maintain excitatory/inhibitory balance in brain tissue; disruption of this balance contributes to disease [86]. In addition, SST regulates Aβ clearance, one of its most important contributions to AD therapy. SST also increases the expression and activity of neprilysin, a major enzyme in the degradation of Aβ peptides [87]. Studies have shown that SST levels are greatly diminished in AD brains, which impairs Aβ degradation and promotes plaque formation. A potential therapeutic strategy is to restore SST levels or enhance its signaling pathways to reduce Aβ burden and slow disease progression. Furthermore, SST plays an important role in both Aβ metabolism and memory function [87]. It controls synaptic plasticity by modulating NMDA receptor activity and increasing LTP, processes that are instrumental for learning and memory. Studies in animals have shown that SST administration can enhance cognitive performance in AD models, suggesting that SST could serve as a therapeutic target in AD. Although SST has potent neuroprotective activity, it is precluded from clinical use because of its short half-life and poor ability to cross the BBB. Currently, researchers are investigating SST analogs and receptor agonists as drug candidates to capitalize on these benefits in treating AD.

Over the past four decades, extensive research has established a strong link between AD and SST. SST and the neurons that express it are critical for normal brain function, particularly in modulating hippocampal activity and memory formation. In AD, the loss of SST and SST-expressing neurons plays a central role in a cascade of interconnected pathological events driven by Aβ, ultimately leading to cognitive decline and dementia. Beyond cognition, the SST system is also implicated in neuropsychiatric symptoms, seizure activity, and neuroinflammation associated with AD. Preclinical studies demonstrate that administration of SST or SST receptor (SSTR) agonists can improve cognition. Notably, activation of SSTR subtype 4 promises in mitigating learning and memory deficits, reducing comorbid symptoms, and enhancing enzymatic breakdown of Aβ in the brain. Overall, targeting SST-mediated pathways offers significant therapeutic potential for treating AD [36].

## 4. Current Advances in Neuropeptide Therapeutics

Section 3 emphasizes the neuroprotective, anti-inflammatory, and synapse-regulating functions of neuropeptides, which has increased interest in developing neuropeptide-based strategies for the treatment of AD. Although encouraging results have emerged from both experimental models and clinical investigations, several limitations still hinder their therapeutic translation, including restricted blood–brain barrier permeability, limited peptide stability, and challenges in achieving targeted delivery within the brain. This section summarizes recent progress in neuropeptide therapeutics, highlighting evidence from preclinical studies, updates from ongoing clinical trials, and the continuing obstacles that must be addressed for successful clinical application (Table 1).

### 4.1. Pituitary Adenylate Cyclase-Activating Polypeptide for AD

Pituitary Adenylate Cyclase-Activating Polypeptide (PACAP) is a neuropeptide with potent neuroprotective properties, making it a promising therapeutic candidate for AD [88]. In vitro studies have demonstrated that PACAP protects hippocampal neurons from Aβ-induced toxicity, a major pathological hallmark of AD [88]. This protective effect is primarily mediated through activation of the cAMP/protein kinase A (PKA) signaling pathway, which enhances neuronal resilience by promoting antioxidant defense mechanisms and mitochondrial stability and by inhibiting apoptosis [88]. PACAP has also been shown to regulate key signaling molecules such as ERK1/2, Akt, and CREB, which contribute to synaptic plasticity and cognitive function. Furthermore, studies using primary cortical and hippocampal neuron cultures have demonstrated that PACAP treatment significantly reduces Aβ-induced oxidative stress and prevents mitochondrial dysfunction, thereby mitigating neuronal death [89].

In vivo studies of transgenic AD models, such as APP/PS1 and 5xFAD mice, have shown that PACAP administration reduces Aβ plaque burden, indicating its potential to modify disease progression. A study using APP/PS1 transgenic mice found that chronic PACAP treatment led to reduced amyloid plaque deposition and improved synaptic plasticity. These effects translated into significant cognitive improvements, as evidenced by enhanced performance in behavioral tests that assess spatial learning and memory [90]. Another key in vivo study demonstrated that PACAP-deficient mice, which lack the PACAP gene, exhibited exacerbated neurodegeneration and memory deficits compared to wild-type controls, supporting PACAP’s critical role in brain homeostasis. These mice showed increased tau hyperphosphorylation, which is linked to neurofibrillary tangle formation, further supporting PACAP’s role in regulating tau pathology.

In a study using hippocampal slice preparations, PACAP was found to enhance long-term potentiation (LTP), a cellular mechanism underlying memory formation [91]. This suggests that PACAP not only protects against neurodegeneration but also actively promotes synaptic repair and cognitive resilience [92]. Another study in aged rats demonstrated that intracerebroventricular PACAP administration reversed age-related cognitive decline by increasing neurogenesis and synaptic density in the hippocampus [93]. Beyond Aβ-related pathology, PACAP has been found to exert anti-inflammatory effects by reducing activation of microglia and astrocytes, which are key contributors to neuroinflammation in AD [93]. A study investigating the effects of PACAP on lipopolysaccharide (LPS)-induced neuroinflammation found that it significantly reduced pro-inflammatory cytokine levels, including TNF-α, IL-6, and IL-1β, further supporting its neuroprotective role [94]. Overall, these findings highlight PACAP as a multifaceted neuropeptide with significant potential in AD therapy. Its ability to reduce amyloid burden, protect against oxidative stress, enhance synaptic plasticity, promote neurogenesis, and modulate neuroinflammation makes it an attractive target for drug development [95]. However, further studies, including clinical trials, are necessary to determine the optimal delivery methods and long-term safety of PACAP-based therapeutics in humans.

### 4.2. Neuropeptide Y (NPY)

NPY is one of the crucial neuropeptides involved in the regulation of neurotransmitter release, synaptic plasticity, and neuroprotection, and it has been recognized as a potential therapeutic target for AD [68]. In vitro, NPY strongly protects neurons from excitotoxic damage by inhibiting glutamate release from presynaptic terminals, thereby preventing excessive NMDA receptor activation. In primary hippocampal and cortical neurons, NPY treatment prevented Aβ-induced synaptic dysfunction and oxidative stress. NPY also regulates intracellular calcium homeostasis, thereby decreasing mitochondrial dysfunction and apoptosis, which are important contributors to AD pathogenesis [82].

Neprilysin (NEP), a key enzyme that degrades Aβ, plays a crucial role in AD pathogenesis. Beyond Aβ degradation, NEP also processes neuropeptides, notably NPY. Studies in transgenic mice overexpressing NEP showed that NEP cleaves NPY into C-terminal fragments (CTFs), primarily NPY 21–36 and 31–36, whereas silencing NEP reduces this processing. Infusion of these NPY CTFs into the brains of APP-transgenic mice ameliorated neurodegenerative pathology, and the amidated CTFs protected human neurons from Aβ toxicity in culture. These findings suggest that NEP’s dual role—both degrading Aβ and generating neuroprotective NPY fragments—contributes to neuroprotection in AD, highlighting a unique multifunctional role for NEP in disease mechanisms [96].

In another study, the effects of NPY were investigated in glutamate-induced excitotoxicity models, and NPY decreased oxidative stress markers and preserved neuronal viability, further supporting its neuroprotective potential study using electrophysiological recordings in hippocampal slices showed that NPY increased long-term potentiation (LTP), a vital process in memory formation, suggesting that NPY may enhance synaptic efficiency in AD-afflicted brains. Apart from its actions on glutamatergic transmission, NPY has also been demonstrated to exert anti-inflammatory effects by modulating microglial activation and reducing pro-inflammatory cytokines that are crucial in AD pathogenesis.

One study demonstrated that NPY provides significant neuroprotection against glutamate-induced excitotoxicity in rat retinal cells both in vitro and in vivo. Glutamate exposure caused necrotic and apoptotic cell death, predominantly in neurons, and this cell death was effectively reduced by NPY pretreatment. The protective effects on necrosis were mediated through activation of NPY Y2, Y4, and Y5 receptors, with the Y5 receptor specifically preventing apoptosis via downstream signaling through protein kinase A (PKA) and p38 kinase (p38K). In vivo, intravitreal NPY injection before glutamate exposure decreased retinal apoptosis and preserved ganglion cells. These findings suggest that targeting NPY receptors, particularly Y5, may offer a promising therapeutic strategy for retinal degenerative diseases such as glaucoma and diabetic retinopathy [97].

NPY, an abundant neurotransmitter in the CNS involved in diverse functions such as cognition and neurogenesis, is reduced in neurodegenerative diseases, including AD. To investigate whether NPY replacement could alleviate AD-related neurodegeneration and behavioral deficits, a lentiviral vector expressing NPY fused to a brain-targeting apoB peptide was delivered broadly to the CNS of APP-transgenic mice. This treatment reversed neurodegenerative and behavioral pathology without affecting Aβ accumulation. Notably, it significantly increased neural precursor cell proliferation in the hippocampal subgranular zone, though without promoting neuronal differentiation. The neuroprotective and neurogenic effects appeared to be mediated by ERK and Akt signaling via NPY receptors Y1 and Y2. These results suggest that widespread CNS delivery of NPY may counteract AD-related neuronal and glial damage and enhance neurogenesis, representing a promising therapeutic strategy, especially in combination with anti-Aβ treatments [98].

A study investigated NPY expression in the mouse hippocampus during early stages following kainic acid (KA)-induced excitotoxicity and its role in neuronal protection. Using immunohistochemistry, NPY expression in the dentate gyrus (DG) granule cell layer appeared 4 h after KA injection, peaked at 8 h, and declined by 16–24 h; in contrast, CA3 showed delayed NPY signals at 16 and 24 h, while CA1 showed none. In situ hybridization revealed NPY mRNA signals in CA3, CA1, and hilus at 4, 8, and 16 h, with strong expression in DG at 4 h that later leveled off. Functional analysis via TUNEL assay demonstrated that intracerebroventricular infusion of NPY and agonists of Y5 and Y2 receptors within 8 h after KA insult significantly rescued pyramidal neurons in CA3 and CA1 from apoptosis. These findings indicate that NPY acts as an important antiepileptic agent by reducing neuronal excitability and preventing excitotoxic apoptosis in a time- and dose-dependent manner through activation of Y5 and Y2 receptors, with early NPY elevation in DG potentially explaining its differential vulnerability compared to CA regions [99].

NPY is a small peptide involved in regulating cardiovascular function, feeding behavior, anxiety, depression, and epilepsy. In the hippocampus, NPY is primarily produced by GABAergic interneurons and inhibits glutamatergic neurotransmission within the excitatory trisynaptic circuit. Under epileptic conditions, NPY and its receptors are markedly upregulated in granule and pyramidal cells, thereby providing tonic inhibition of glutamate release and limiting the spread of excitability across brain regions. Beyond controlling excitability, NPY has recognized neuroprotective effects against excitotoxicity and modulates neurogenesis. Although recent patents focus on NPY receptor antagonists for obesity and cardiovascular diseases, the NPY system also shows promise as a target for developing new antiepileptic therapies and brain repair strategies. Future work should aim to design selective NPY receptor agonists that effectively reach their targets in the epileptic brain, thereby harnessing these therapeutic benefits [100].

In organotypic mouse hippocampal slice cultures exposed to the glutamate receptor agonist AMPA, NPY receptor activation conferred a neuroprotective effect against excitotoxicity-induced degeneration. Specifically, 24 h exposure to 8 µM AMPA caused significant degeneration of CA1 and CA3 pyramidal neurons, as indicated by increased propidium iodide (PI) uptake. Treatment with a Y2 receptor agonist [NPY (13–36), 300 nM] significantly reduced neuronal damage in these regions, and this effect was blocked by a selective Y2 receptor antagonist (BIIE0246) but was unaffected by inhibition of BDNF signaling. Additional experiments showed that blocking Y1 receptors or neutralizing endogenous NPY unmasked a neuroprotective role of NPY in CA1. AMPA exposure also increased BDNF levels, upregulated neuronal TrkB receptors, and induced BDNF-expressing microglia at injury sites. Together, these results suggest that activation of Y1 and Y2 NPY receptors triggers neuroprotective pathways in the hippocampus that mitigate AMPA-induced neurodegeneration, with BDNF released from neurons and microglia potentially modulating these effects [101].

Research over recent decades has identified NPY neurons in the arcuate nucleus (Arc) of the hypothalamus as key regulators of feeding behavior, acting as powerful stimulators of food intake. However, advances using genetic mouse models alongside chemogenetic and optogenetic tools have revealed that these Arc NPY neurons have broader roles beyond simply promoting hunger. They are involved in controlling energy expenditure, thermogenesis, physical activity, food-seeking behavior, and anxiety. This diverse regulation occurs through complex neural networks linking Arc NPY neurons to other critical brain regions such as the paraventricular nucleus, ventral tegmental area, amygdala, and brainstem. Furthermore, single-cell sequencing has uncovered considerable heterogeneity within NPY neurons, identifying distinct subpopulations characterized by co-expression of various neurotransmitters [102].

NPY system protects against immune challenges by reducing sickness behavior and preventing the development of depression, thus maintaining homeostasis. NPY serves as a crucial signaling molecule that coordinates interactions between the immune system and brain function in both health and disease [103].

A study identified NPY as a crucial modulator of inflammation-associated microglial motility, acting through Y1 receptor activation to inhibit lipopolysaccharide (LPS)-induced movement in microglial cells (N9 line). The involvement of endogenous interleukin-1 beta (IL-1β) was supported by the observation that LPS-stimulated microglial motility was reduced by an IL-1 receptor antagonist. Direct IL-1β stimulation promoted p38 mitogen-activated protein kinase (MAPK) activation and increased microglial motility, while inhibition of p38 MAPK reduced actin reorganization linked to cell movement. Importantly, NPY suppressed p38 phosphorylation via Y1 receptors, thereby limiting microglial motility. This inhibitory effect of NPY on LPS-induced motility was also confirmed in mouse brain cortex explants. Overall, these findings reveal a novel role for NPY in regulating microglial behavior and may be significant for controlling microglial migration in central nervous system injuries and diseases [104].

NPY is extensively distributed throughout the central nervous system, where it regulates numerous vital physiological processes and contributes to various pathological conditions, including obesity, anxiety, epilepsy, chronic pain, and neurodegenerative disorders [105].

A recent study investigated whether NPY confers neuroprotection in AD models and examined the involvement of neurotrophins in NPY-mediated effects in SH-SY5Y neuroblastoma cells. The study found that preincubation with NPY significantly prevented Aβ25–35-induced cell loss. Whereas Aβ exposure reduced neurotrophin protein and mRNA levels, NPY treatment restored or elevated their expression in SH-SY5Y cells. These findings suggest that NPY enhances neuronal survival and counteracts β-amyloid toxicity by restoring neurotrophin levels, highlighting NPY’s potential role in advancing the understanding of AD pathology and guiding therapeutic development [37].

NPY is highly concentrated in the cerebral cortex and present in cortical neurons, was measured in postmortem brain tissue from AD patients and controls using a sensitive radioimmunoassay. High-performance liquid chromatography confirmed that over 95% of the detected immunoreactivity corresponded to authentic NPY in both groups. Significant reductions in NPY-like immunoreactivity were observed in 11 cortical regions, including the hippocampus and locus ceruleus, with particularly pronounced decreases in the temporal, frontal, and occipital lobes. Given that NPY is co-localized with somatostatin in many cortical neurons, this loss likely reflects degeneration of these neuronal populations. Investigating the selective vulnerability of these neurons in AD may offer important insights into the disease’s underlying mechanisms [106].

NPY promotes neurogenesis in the subventricular zone (SVZ), a key source of neurons for brain repair and cell replacement therapies. In SVZ cell cultures, treatment with NPY (1 μM) significantly increased cell proliferation at 48 h and neuronal differentiation at 7 days. These proneurogenic effects are mediated primarily by the Y1 receptor, the predominant functional NPY receptor in the mouse SVZ, as demonstrated by functional autoradiography. Additionally, brief NPY exposure enhanced nuclear immunoreactivity of phosphorylated extracellular signal-regulated kinase 1/2 (ERK1/2), indicating stimulation of proliferation. In contrast, longer treatment (6 h) increased phosphorylated c-Jun N-terminal kinase (JNK) signaling in growing axons, supporting axonogenesis. Thus, NPY acts as a critical regulator of SVZ neurogenesis, highlighting its potential for advancing cell-based brain therapies [106].

A study identified early, selective neurodegeneration of dendritic inhibitory interneurons—specifically oriens-lacunosum moleculare (O-LM) and hilar perforant path-associated (HIPP) cells—in the hippocampus of a transgenic PS1 × APP AD mouse model. At 6 months, among 22 synaptic markers tested, only somatostatin (SOM) and neuropeptide Y (NPY), which mark these interneurons, showed significant decreases. Stereological analysis revealed a 50–60% loss of SOM-positive neurons, preceding pyramidal neuron loss, with the subset co-expressing NPY being most affected. A strong linear correlation was found between SOM/NPY deficiency and amyloid-β accumulation. These findings suggest early hippocampal pathology in AD and highlight SOM and NPY neuropeptides as potential early biomarkers for monitoring treatment efficacy [107].

NPY agonists inhibit glutamate release at the rat hippocampal CA3–CA1 synapse by acting presynaptically on Y2 receptors, which reduce calcium influx through voltage-dependent calcium channels (VDCCs). Using optical measurements with the calcium indicator fura-2 and electrophysiological recordings, investigators showed that Y2 receptor activation decreases presynaptic calcium entry by inhibiting multiple types of VDCCs, including N-type, P/Q-type, and others. This reduction in calcium influx thereby suppresses synaptic transmission. Additionally, activation of adenosine receptors fully occludes the Y2 receptor-mediated inhibition of calcium influx, indicating that both receptors likely converge on the same G-protein signaling pathway, whereas GABAB receptor-mediated inhibition acts independently [108].

A study investigated the role of NPY Y4 receptors in emotional behavior by comparing female Y4 receptor knockout (Y4−/−) mice with control mice and Y2 receptor knockout (Y2−/−) mice. Both Y4−/− and Y2−/− mice showed reduced anxiety-like behavior and increased locomotor activity in anxiety tests (open field and elevated plus maze) compared with controls. Whereas Y4−/− mice maintained normal locomotion in familiar environments, Y2−/− mice showed reduced activity. Both knockouts altered daily body temperature rhythms and affected stress-induced hyperthermia differently depending on time and genotype. In the tail suspension test of depression-like behavior, Y4−/− and Y2−/− mice displayed less immobility, indicating reduced depression-like symptoms. However, only Y2−/− mice showed impaired object recognition memory, whereas Y4−/− mice did not. These findings suggest that Y4 receptor deletion, like Y2 receptor deletion, reduces anxiety and depression-like behaviors but does not impair memory, highlighting an important role for Y4 receptors in behavioral regulation and homeostasis [109].

A recent study demonstrated that NPY protects neurons by modulating microglial activity and neuronal excitability. When rat cortical microglia were activated with LPS, they produced increased levels of the pro-inflammatory cytokines IL-1β and TNF-α. Conditioned medium from these activated microglia increased NMDA receptor-mediated currents (INMDA) in cultured cortical neurons, which can lead to excitotoxicity. However, treatment with NPY suppressed both cytokine production in microglia and the elevated INMDA in neurons. The protective effect of NPY was fully blocked by a Y1 receptor antagonist, indicating that NPY acts through Y1 receptors to inhibit microglial activation and reduce excitotoxic NMDA currents, ultimately protecting neurons from damage [75].

AD, a prevalent neurodegenerative disorder, may involve changes in NPY, which participates in adult neurogenesis, immune regulation, the inhibition of voltage-dependent Ca^2+^ channels, and the reduction in glutamate excitotoxicity. A systematic review analyzed 19 studies exploring NPY’s involvement in AD and its potential as a biomarker and therapeutic target. Findings on NPY levels in cerebrospinal fluid and plasma from patients with AD were inconsistent—some studies reported reductions, whereas others found no change—highlighting the need for further research. However, data from transgenic animal models and in vitro and in vivo experiments support NPY’s neuroprotective role in AD pathogenesis. These insights suggest that NPY could serve as an early diagnostic marker and a promising therapeutic target to counteract neurodegeneration in AD [110].

### 4.3. Vasoactive Intestinal Peptide (VIP)

VIP is critical for neuronal survival, reduces oxidative stress, and improves cognitive function, all of which are disrupted in AD [57]. In vitro, VIP has been shown to protect neurons from Aβ-induced oxidation and mitochondrial dysfunction, which are major pathways of neurodegeneration in AD. For instance, VIP can prevent Aβ-mediated mitochondrial impairment by enhancing antioxidant enzyme activity, leading to decreased ROS accumulation, as demonstrated in studies performed on hippocampal and cortical neuron cultures. Furthermore, VIP has been demonstrated to inhibit pro-apoptotic pathways, thereby preventing neuronal death and maintaining cell viability under AD-like conditions [57,111].

### 4.4. Somatostatin (SST)

Rofo et al. addressed the challenge of Aβ aggregation in AD by enhancing Aβ degradation through neprilysin activation by somatostatin (SST). Because SST levels are reduced in AD brains and its therapeutic use is limited by poor blood–brain barrier (BBB) penetration and an approximately 3 min half-life, the researchers engineered a fusion protein, SST-scFv8D3, combining SST with a BBB transporter targeting the transferrin receptor. This fusion protein exhibited a 120-fold longer half-life than SST alone and effectively penetrated the brain after intravenous injection. In APPswe transgenic mice overexpressing mutant amyloid precursor protein, SST-scFv8D3 administration significantly elevated neprilysin levels and selectively reduced membrane-bound Aβ42 in the hippocampus and adjacent cortex after three injections. Given that membrane-bound Aβ42 is highly neurotoxic and the hippocampus is critically involved in AD progression, this BBB-permeable SST fusion represents a novel therapeutic approach with a promising safety profile that targets disease-relevant brain regions in AD [112].

Another study investigated the role of inhibitory interneurons in AD, focusing on two major subtypes: parvalbumin (PV) and somatostatin (SST) interneurons. Using immunohistochemistry on postmortem brain tissue from AD patients and controls, the researchers examined these interneurons in the temporal cortex and hippocampus. They found a higher density of both PV and SST interneurons in the temporal cortex than in the hippocampus. Importantly, AD cases showed a significant, selective reduction in SST, but not PV, interneurons in the temporal cortex relative to controls. These findings indicate a region-specific vulnerability of SST interneurons in AD and suggest that disruption of this inhibitory circuit may contribute to neuronal dysfunction and the cognitive decline characteristic of the disease [113].

A related study revealed a novel mechanism by which SST regulates amyloid β (Aβ) degradation in AD. The researchers identified α-endosulfine (ENSA), an endogenous ligand of ATP-sensitive potassium (KATP) channels, as a negative regulator of neprilysin (NEP), the key enzyme upregulated by SST to degrade Aβ. ENSA levels are elevated in AD mouse models and patients, and NEP itself degrades ENSA, thereby creating a feedback loop that controls NEP activity. The study further identified a specific KATP channel subtype that modulates NEP function and affects Aβ accumulation. Pharmacologically targeting this KATP channel subtype reduced Aβ deposition and improved memory in AD mice through NEP activation. These findings uncover a molecular link between KATP channel signaling and NEP-mediated Aβ clearance, offering potential therapeutic avenues for AD prevention [114].

In another study, amyloid β oligomers (AβO) in AD were shown to impair hippocampal theta and gamma oscillations, which are crucial for memory. These rhythms rely on somatostatin-positive (SST) and parvalbumin-positive (PV) interneurons, respectively. Using optogenetics in AβO-injected mice, the authors showed that activation of SST and PV interneurons selectively restored the reduced power of theta and gamma oscillations and resynchronized pyramidal neuron firing in the hippocampal CA1 region. Electrophysiological recordings showed that AβO increased the initial GABA release probability but depressed inhibitory inputs from SST and PV interneurons to CA1 pyramidal cells at theta and gamma frequencies, respectively, indicating presynaptic dysfunction. These findings reveal frequency- and interneuron subtype-specific synaptic deficits caused by AβO and suggest that targeting SST and PV interneuron dysfunction could be a therapeutic strategy for restoring hippocampal network oscillations and cognitive function in early AD [115].

In an experimental study, exosomes were engineered to express brain-targeting ligands—either cholecystokinin (CCK) or somatostatin (SST)—along with CD47 to reduce immune clearance. MicroRNA-29b-2 (miR29b-2), known to reduce presenilin 1 (PSEN1) expression and β-amyloid accumulation, was loaded into these exosomes. In exosomes produced from gene-modified dendritic cells, CD47-SST exosomes achieved superior brain delivery efficiency compared with CD47-CCK exosomes after intravenous administration in mice. Furthermore, miR29b-2-loaded CD47-SST exosomes effectively reduced PSEN1 protein levels and inhibited β-amyloid oligomer production in both cellular and 3xTg-AD mouse models. These results demonstrate the potential of engineered exosomal nanocarriers for precise, immune-evasive delivery of oligonucleotide drugs to the brain, offering a promising platform for AD treatment and other diseases [115].

Another study explored the neuroprotective role of somatostatin-14 in differentiated SH-SY5Y cells exposed to Aβ-induced toxicity. SST enhanced cell viability and mitochondrial stability while modulating apoptotic pathways activated by Aβ. It inhibited phosphorylation of Collapsin Response Mediator Protein 2 (CRMP2) at Ser522, a modification primarily driven by cyclin-dependent kinase 5 (CDK5). Additionally, SST regulated intracellular calcium (Ca^2+^) influx and suppressed calpain activation, both key mediators of neurotoxicity, through the somatostatin receptor subtype 2 (SSTR2). Notably, SST’s effects on Ca^2+^ homeostasis and calpain activity appeared independent of CDK5 expression and p35/25 accumulation. These findings reveal two distinct mechanisms by which SST mediates neuroprotection—through regulation of calcium signaling and CRMP2 phosphorylation—highlighting its therapeutic potential in AD and other neurodegenerative disorders where calcium dysregulation plays a critical role [116].

Growing evidence now highlights the potential of targeting somatostatin-mediated Aβ catabolism in the brain through the α-endosulfine (ENSA)–KATP channel pathway as a promising therapeutic strategy. This pathway offers an opportunity for drug repurposing by utilizing existing FDA-approved drugs indicated for other conditions to modulate this mechanism. Advances in biomedical technologies, including in silico modeling and in vitro assays, enable deeper exploration and validation of this complex pathway. An article by Varghese et al. reviews the α-endosulfine–KATP channel signaling axis and discusses its potential to guide future AD therapy development [116].

AD, which is characterized by Aβ peptide aggregation, may be therapeutically targeted by enhancing Aβ degradation through activation of the enzyme neprilysin. The SST peptide, particularly the brain-penetrating variant SST-scFv8D3, has been shown to increase neprilysin activity and promote Aβ42 degradation in the hippocampus of APPswe transgenic mice. Proteomic analysis via LC–MS revealed that treatment with SST-scFv8D3 downregulated mitochondrial proteins involved in fatty acid oxidation—proteins typically elevated in APPswe mice—while upregulating synaptic proteins related to membrane trafficking and neuronal development. Additionally, hippocampal levels of the growth-regulated α (KC/GRO) chemokine and the degradation of neuropeptide Y increased following treatment. Together, these findings suggest that SST-scFv8D3 exerts multifaceted effects on mitochondrial regulation and neurogenesis, supporting the potential of SST peptide-based therapies for AD [117,118].

SST also plays a novel role in modulating long-term potentiation (LTP) at excitatory synapses on somatostatin-expressing interneurons (SOM-INs) in the CA1 hippocampus. While SST’s effects on pyramidal cells are well known, its impact on inhibitory interneurons is less clear. Using pharmacological tools and recordings from transgenic mice, researchers found that exogenous SST14 induces LTP of excitatory postsynaptic potentials specifically in SOM-INs via somatostatin receptors (SST1–5Rs), without affecting pyramidal cells or parvalbumin interneurons. This Hebbian LTP was prevented by blocking SST receptors or depleting endogenous SST, indicating SST’s critical role in this process. The SST-induced LTP was independent of NMDA and mGluR1a receptor activation but required activity and was blocked by GABAA receptor antagonism, suggesting that SST facilitates LTP by modulating GABAergic inhibition. Overall, endogenous SST contributes to synaptic plasticity in SOM-INs, highlighting a previously unrecognized mechanism important for hippocampus-dependent learning and memory [119].

## 5. Clinical Trials Involving Neuropeptide-Based Drugs

Recently, neuropeptide-based drugs have attracted considerable attention because they can exert multiple effects that modulate the progression of AD, including Aβ clearance, a reduction in neuroinflammation, an enhancement in synaptic plasticity, and the modulation of neurotransmission; however, clinical data are limited for these neuropeptides [72].

VIP analogs have been used in clinical trials for AD as VIP has neuroprotective and anti-inflammatory activity. In a previous trial, administration of VIP analogs to patients with AD yielded preliminary evidence of improved cognitive function and reduced markers of neuroinflammation, suggesting that VIP analogs might slow disease progression [120]. Another clinically relevant neuropeptide, PACAP, has been investigated for its neurotrophic and anti-apoptotic properties. In a small-scale trial in patients with early-stage AD, intravenous administration of PACAP improved memory retention and reduced markers of oxidative stress, supporting a role for PACAP in promoting neuronal survival. Additional trials are needed to define the long-term benefits and optimal dosing regimens [62,121]. A second major clinical study examined NPY-based therapy, motivated by its effects on synaptic plasticity and its protective action against excitotoxicity. In patients receiving intrathecal NPY, executive function and working memory improved, and tau hyperphosphorylation markers in cerebrospinal fluid decreased. NPY may therefore oppose key pathological hallmarks of AD [98]. SST analogs have been studied in patients with AD in a separate clinical trial because SST is involved in Aβ clearance and cognition. In that trial, patients received a long-acting SST analog by subcutaneous injection and showed slowed cognitive decline and increased cerebrospinal fluid levels of neprilysin, an enzyme that degrades Aβ [122]. These data support the hypothesis that restoring SST signaling could provide a therapeutic option. Another potentially fruitful approach involves SP receptor antagonists, as SP is associated with neuroinflammation and glial activation [123]. Targeting SP pathways by surgically blocking the SP receptor in AD model studies has been shown to reduce infiltration of peripheral immune cells into the brain, suggesting a possible strategy to mitigate neuroinflammation-associated damage in AD. Consistently, a clinical study indicated that SP receptor blockade reduced neuroinflammatory markers and slightly improved certain cognitive tests in patients with mild-to-moderate AD [124].

**Figure 5:**
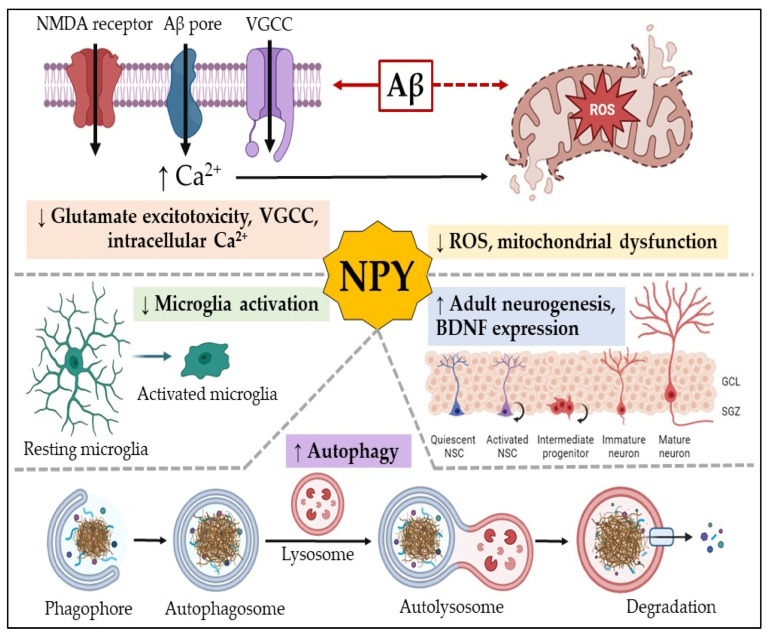
Neuropeptide Y (NPY) protects against Alzheimer’s disease caused by Aβ by lowering calcium overload, oxidative stress, and neuroinflammation. It also encourages neurogenesis, BDNF expression, and neuronal autophagy to help cells stay alive [126].

Patients receiving dual-neuropeptide therapy had greater cognitive benefits than those receiving either agent alone, suggesting a synergistic effect (Figure 5). While these clinical investigations reveal multiple potential avenues for the use of neuropeptides in AD therapy, additional research is needed to optimize their clinical use, refine dosing strategies, and address challenges related to drug delivery and BBB penetration [124]. Neuropeptide-based drugs and their therapeutic targets are presented in Table 2.

**Table 2:** Status of Neuropeptide-Based Drugs and Their Therapeutic Targets.

Neuropeptide-Based Drugs	Target Disease	Status	Reference
Cerebrolysin	mild-to-moderate Alzheimer’s disease	marketed	Gauthier et al. [125]
Davunetide (AL-108) (NAP)	Tauopathies	Under clinical trials	NCT01056965

## 6. Potential Strategies for Neuropeptide Drug Delivery

### 6.1. Nanocarrier-Based Delivery Systems

Effective delivery of neuropeptide-based drugs for AD is hindered by the BBB, enzymatic degradation, and rapid clearance from circulation. Despite these barriers, liposomes, nanoparticles, and polymeric micelles are being investigated as nanocarrier-based delivery systems to overcome limitations in stability, bioavailability, and targeted brain delivery of these compounds [126,127,128,129,130,131,132,133,134,135,136,137,138]. Among these, liposomes—biocompatible phospholipid vesicles—have been extensively studied for neuropeptide delivery because they can encapsulate both lipophilic and hydrophilic molecules and protect them from enzymatic degradation. Interestingly, in AD, delivery of PACAP and VIP using a liposome-based delivery system has resulted in improved neuroprotection, greater bioavailability, and a prolonged half-life [139]. Intranasal administration of PACAP-loaded liposomes via the olfactory and trigeminal pathways, bypassing the BBB, significantly improves cognitive function and decreases Aβ accumulation in AD models. Plastic nanoparticles, including PLGA, chitosan, and PCL, are another highly promising nanocarrier system, as they may provide controlled and sustained drug release and enhance neuropeptide stability [140]. NPY encapsulated in PLGA nanoparticles has been shown to cross the BBB and reach neurons in the hippocampus and prefrontal cortex, where it enhances synaptic plasticity and neuroprotection in AD models [141]. Studies have shown that VIP-conjugated gold nanoparticles, when further conjugated with transferrin, successfully cross the BBB via receptor-mediated transcytosis and exert potent anti-inflammatory and neuroprotective effects in AD models. This makes exosomes an excellent vehicle for neuropeptide-based therapeutics, as they are the only carriers that can efficiently cross the BBB and deliver their cargo directly to neuronal cells. Moreover, lipid nanocapsules and solid lipid nanoparticles are being explored as potential strategies for prolonging drug circulation time, protecting neuropeptides from enzymatic degradation, and enabling targeted brain delivery. Promising results have been obtained from the intranasal administration of SLNs encapsulating OXT and VIP in improving cognitive deficits, reducing neuroinflammation, and enhancing synaptic plasticity in AD models [142]. Furthermore, the surfaces of nanoparticles are being modified with ligands that penetrate the BBB, including transferrin, insulin, and lactoferrin, to increase the efficiency of neuropeptide delivery. Consequently, lactoferrin-functionalized nanoparticles loaded with PACAP crossed the BBB more efficiently and improved memory function in AD rat models compared with nanoparticles without lactoferrin functionalization [143]. However, delivering neuropeptides as drugs for AD therapy is challenging because of their poor solubility, rapid degradation, and low permeability across the BBB, and delivery systems specifically designed for neuropeptides in AD have rarely been reported. Nevertheless, optimal formulations still need to be developed, and long-term safety and translation to human clinical trials should be evaluated to establish their efficacy in the treatment of AD [143].

### 6.2. Peptide Analogs and Modifications for Increased Stability

Neuropeptides are often limited by their short half-life, susceptibility to enzymatic degradation, and poor BBB penetration, which substantially constrains their clinical potential for the treatment of AD [72,144]. Overcoming these challenges has led researchers to synthesize peptide analogs and to modify peptide structures in the hopes of improving stability, bioavailability, and receptor selectivity while retaining their neuroprotective properties [145]. The most effective methods are N-terminal and C-terminal modifications that replace or modify amino acid residues to confer resistance to enzymatic cleavage [146]. For instance, the PACAP analog Ac-PACAP27-NH2 has shown improved resistance to dipeptidyl peptidase IV (DPP-IV) and neprilysin, along with significantly greater neuroprotection and cognitive benefits in an AD model. Likewise, researches have aimed to increase the membrane permeability, metabolic stability, and potential peripheral actions of VIP analogs such as Stearyl-NH-VIP and its derivatives through systemic administration [147].

Cyclization is another widely used approach to help limit peptide flexibility and, thus, prevent enzymatic degradation. With regard to cyclic analogs, disulfide-bridge and lactam-based cyclization of NPY and SST analogs have been successfully employed to increase their resistance to proteolysis and enhance receptor selectivity [148]. For example, cyclized SST analogs, including octreotide and pasireotide, have retained their neuroprotective and anti-amyloidogenic properties and therefore could be promising candidates for AD treatment. β-sheet breaker peptides have also been chemically modified by D-amino acids, peptoid backbones, and hydrocarbon stapling to improve their stability and prolong therapeutic action [149]. Substitution with D-amino acids, which replaces an L-amino acid with the respective D-enantiomer, has been particularly effective for inhibiting proteolytic degradation. Studies have shown that D-amino-acid-modified PACAPs and VIPs maintain their biological activity while exhibiting increased resistance to enzymatic degradation and a prolonged plasma half-life [67]. Furthermore, peptidomimetics and backbone modifications have been employed to improve both neuropeptide function and pharmacokinetics. Peptidomimetics are typically structurally modified peptides with non-natural backbones designed to have greater metabolic stability and BBB permeability than their native counterparts [150]. For example, small molecules that are mimetics of PACAP and peptidomimetics based on VIP have been synthesized to maintain receptor activation without the rapid enzymatic breakdown of the ligand. PEGylation, the attachment of polyethylene glycol (PEG) chains to peptides to increase solubility, decrease renal clearance, and prolong systemic circulation time, is another innovative approach [151]. PEGylating NPY and OXT analogs has resulted in significantly improved pharmacokinetics, sustained receptor activation, and enhanced cognitive benefits in AD models [135]. Substantial effort has also focused on lipidation—the attachment of fatty acid chains to neuropeptides—to increase membrane permeability, receptor binding, and bioavailability. Another strategy involves fusion peptides, in which neuropeptides are linked to carrier proteins or cell-penetrating peptides (CPPs) to facilitate transport across the BBB [152]. TAT-conjugated PACAP and NPY analogs exhibit significantly increased brain uptake as well as enhanced neuroprotective effects vs. their unmodified counterparts [153]. Overall, peptide analogs and modifications represent important advances toward neuropeptide-based therapeutics for AD. Multiple approaches—including chemical modifications, backbone engineering, lipidation, PEGylation, cyclization, and peptidomimetic design—have successfully improved neuropeptide stability, BBB penetration, and therapeutic efficacy. These modifications increase the half-life and bioactivity of neuropeptides, improve their target specificity and functional effects, and make them potential candidates for future clinical applications in AD treatment [154].

### 6.3. Gene Therapy and Neuropeptide Receptor Modulation

Modulating neuropeptide production, receptor expression, and downstream signaling pathways represent an innovative approach to enhance neuropeptide-based therapeutics in AD using gene therapy and neuropeptide receptor modulation [155]. In gene therapy, genetic material is delivered to neurons to increase or restore neuropeptide levels, whereas in receptor modulation, neuropeptide receptors are activated or inhibited to regulate neuronal function, synaptic plasticity, and neuroinflammation [156]. Viral vectors, including adeno-associated viruses (AAVs) and lentiviruses, are among the most promising tools in gene therapy because they enable the introduction of genes encoding neuropeptides and their receptors into the brain [157]. Studies have demonstrated that AAV-mediated delivery of PACAP or its receptor PAC1 exerts ameliorative effects in AD models, including enhanced PACAP signaling, reduced Aβ accumulation, and improved cognitive function [158]. Likewise, gene therapy targeting VIP and its receptor VPAC2 has shown robust anti-inflammatory and neuroprotective effects in preclinical AD models [159].

CRISPR-Cas9 is another powerful gene therapy platform for modulating neuropeptide receptors by inducing overexpression or correcting gene mutations that disrupt neuropeptide signaling. For example, CRISPR activation of NPY receptors Y1 and Y2 has reduced excitotoxicity, improved synaptic plasticity, and enhanced memory formation in AD models [160].

An additional important strategy is direct neuropeptide receptor modulation using agonists, antagonists, and allosteric modulators to fine-tune receptor activity. Stimulation of PAC1 receptors by the PAC1 receptor agonist maxadilan and synthetic PACAP analogs may enhance neurogenesis, support synaptic repair, and prevent tau hyperphosphorylation [161]. Similarly, VPAC2 receptor agonists exert potent anti-inflammatory effects through microglial deactivation and reductions in oxidative stress in AD models. Agonists of NPY receptors (Y1 and Y2) with selectivity for these targets have been tested for the restoration of synaptic plasticity and protection against glutamate-induced excitotoxicity, a major contributor to neuronal loss in AD [146]. Modulation of SST receptors has also attracted interest as a means to enhance memory and Aβ degradation. SST4 receptor agonists have been shown to increase neprilysin activity, one of the key enzymes involved in Aβ clearance, and to decrease plaque burden in AD mouse models [162].

**Figure 6:**
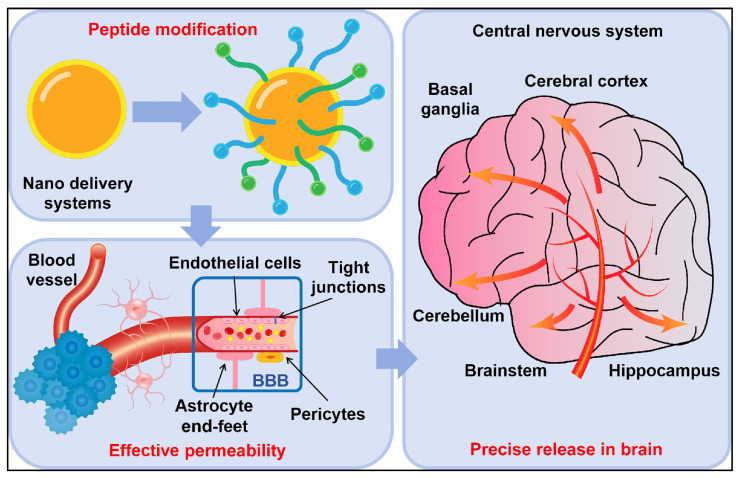
Schematic illustration of neuropeptide-based drug delivery system for targeted CNS delivery. Figure is adopted from [Qian Y et al. [167]].

The short half-life and rapid degradation of peptide-based drugs pose major challenges for neuropeptide receptor modulation (Figure 6). To address this, researchers have developed long-acting peptide analogs and small-molecule modulators of neuropeptide receptors with improved stability and blood–brain barrier (BBB) penetration [162]. For example, to mimic PACAP’s neuroprotective effects while avoiding rapid proteolytic degradation, a series of non-peptidic PAC1 receptor agonists have been designed. Similarly, biased agonists and allosteric modulators of neuropeptide receptors preferentially activate beneficial signaling pathways while minimizing activation of pathways associated with adverse effects [163]. For instance, biased PAC1 receptor agonists may favor activation of pro-survival ERK1/2 signaling while limiting excessive cAMP activation and thereby reducing receptor desensitization. Neuropeptide receptor modulation and gene therapy together could enable long-term, highly specific treatments for AD [164]. Genetic augmentation of neuropeptide production or precise receptor tuning may reverse neurodegeneration, reduce Aβ and tau pathology, and restore cognitive function. However, substantial challenges remain, including immune responses to viral vectors, off-target effects of gene editing, and complexities inherent to neuropeptide receptor signaling, which may be highly specialized within specific neuronal circuits [165]. Beyond sustaining neuropeptide expression via gene therapy, combining gene therapy with receptor modulation may provide a synergistic strategy to achieve precise control of receptor activity and support the development of next-generation neuropeptide-based therapeutics for AD [166].

## 7. Future Perspectives and Research Directions

Despite growing evidence supporting the involvement of neuropeptides such as PACAP, VIP, Substance P, Neuropeptide Y, Somatostatin, and CRF in AD pathophysiology, their translation into therapeutic strategies remains in its infancy. These neuropeptides offer promising avenues due to their diverse regulatory roles in neuroinflammation, oxidative stress, synaptic plasticity, circadian rhythm, and stress responses—processes intricately linked with AD progression. In the coming years, PACAP and VIP will hold significant potential as multifunctional neuroprotective agents, particularly due to their anti-apoptotic, anti-inflammatory, and neurotrophic properties [168]. However, challenges such as their short half-lives, poor BBB penetration, and peptide degradation must be addressed through novel delivery systems (e.g., nanoparticle carriers, intranasal administration, or peptide analogs).

SP and CRF are closely linked to neuroinflammatory and stress-related pathways. While their modulation could help reduce chronic neuroinflammation and HPA axis dysfunction, context-dependent effects and receptor subtype specificity present key hurdles. Broad-spectrum antagonism may lead to undesirable systemic effects [169].

NPY and SST are also of considerable interest due to their roles in neurogenesis, energy metabolism, and their direct interactions with amyloidogenic pathways. In particular, SST has been linked to the regulation of neurolysin, a critical Aβ-degrading enzyme. However, age-related decline in these peptides and the lack of highly selective agonists/antagonists challenge their clinical utility.

Neuropeptide-based therapeutics for AD are highly promising, and ongoing research focuses on identifying emerging neuropeptide targets, developing combination therapies with current AD treatments, implementing AI-driven drug discovery, and advancing personalized medicine [170]. As our understanding of neuropeptide signaling deepens, new targets are being recognized that may enhance neuroprotection, modulate synaptic plasticity, and reduce neuroinflammation. Recently, neuropeptides including corticating (CST), galanin (GAL), and SP have received attention for their roles in cognitive function, neuroinflammation, and neuronal survival. For example, inhibition of pro-inflammatory cytokine release reduces neuroinflammation and Aβ toxicity, and GAL regulates hippocampal neurotransmission and exerts neuroprotective effects. Such emerging neuropeptide targets warrant further exploration for the discovery of novel therapeutic agents that offer multiple therapeutic benefits in the management of AD [171].

Because the pathophysiology of AD is highly complex, future research on neuropeptide-based drugs for AD should focus on combination therapies with currently available AD treatments. Traditional treatments such as cholinesterase inhibitors (donepezil, rivastigmine) and NMDA receptor antagonists (memantine) provide only symptomatic relief and do not slow disease progression. Neuropeptide-based therapeutics could be combined with these agents to enhance therapeutic efficacy. For example, VIP or PACAP could be co-administered with memantine to reduce excitotoxicity and simultaneously induce neuroprotection. Moreover, targeting neuroinflammation through NPY or OXT together with anti-amyloid monoclonal antibodies (i.e., aducanumab or lecanemab) may have synergistic effects, simultaneously preserve cognitive function and reducing amyloid burden. Because AD is a multifactorial disease, combination strategies are needed for optimal control; therefore, multi-targeted future clinical trials should be considered [167].

Within the emerging frontier of neuropeptide therapeutics, AI is used to drive drug discovery and accelerate research and development. Artificial intelligence (AI) and machine learning (ML) algorithms can analyze large volumes of data to identify novel neuropeptide sequences, anticipate their receptor interactions, and, with the recently decreasing costs of synthesis, tune their pharmacokinetics. AI can also be used to design peptide analogs that have better BBB penetration, receptor specificity, and stability. For instance, deep learning models have been utilized to modify PACAP analogs to make them more resistant to enzymatic degradation while maintaining their neuroprotective functions. Furthermore, AI-based virtual screening and molecular docking studies can help in identifying new peptide-based compounds that might have therapeutic use in AD. With these AI-based approaches, drug development timelines will be significantly reduced, and promising leads will be rapidly advanced to clinical testing [172].

Finally, neuropeptide therapeutics in AD must align with the evolving paradigm of personalized medicine. AD is highly heterogeneous, with variability in disease progression, genetic susceptibility, and response to treatment. The goal of precision medicine is to tailor treatments based on a person’s unique genetic makeup, biomarker levels, and disease stage. Neuroimaging, CSF biomarkers, and pharmacogenomic profiling can help identify patient subgroups that would most benefit from targeted neuropeptide-based therapies. Moreover, real-time monitoring of neuropeptide levels using advanced biosensors could facilitate optimization of dosing regimens and enhance therapeutic success [173].

Although important challenges such as stability, BBB penetration, and receptor specificity remain, neuropeptide-based drugs ultimately need to be translated into clinical use. Overall, progress in this field will be driven by interdisciplinary collaborations involving neuroscientists, medicinal chemists, AI researchers, and clinicians to develop the next generation of neuropeptide-based therapeutics, which hold great potential to overcome AD. A holistic approach to this neurodegenerative disorder—integrating emerging targets, combination therapy strategies, AI-driven drug discovery, precision medicine, and related advances—offers hope for more effective treatments in the near future [174].

## 8. Scientific Challenges in Targeting Neuropeptides for AD

Neuropeptide-based therapies face several challenges that limit their clinical application, particularly for AD (Table 3). These peptides, such as PACAP, VIP, and SST, have short half-lives and are rapidly degraded in vivo, resulting in low plasma and CNS stability and necessitating the development of stabilized analogs. Their poor penetration across the BBB further hinders therapeutic delivery, often requiring alternative approaches such as intranasal administration or nanocarriers. Additionally, neuropeptides have pleiotropic systemic effects, raising concerns about off-target actions and making precise, brain region-specific modulation difficult [9]. The complexity and redundancy of their GPCRs add to this challenge, complicating the development of selective ligands. An incomplete understanding of their signaling pathways in AD also limits rational drug design and biomarker identification. Moreover, their effects can be context-dependent and bidirectional, varying with disease stage, receptor type, and brain region, which increases the risk of unintended pathological effects. The translational gap is further widened by a lack of clinical trials and over-reliance on animal models that may not accurately reflect human AD pathology. Finally, neuropeptides often interact with classical neurotransmitter systems, necessitating integrated, systems-level approaches to fully understand their roles in neural network dysfunction associated with AD [175].

## 9. Conclusions

The neuropeptides examined in this article—PACAP, VIP, Substance P, Neuropeptide Y, Somatostatin, and CRF—constitute a heterogeneous array of signaling molecules that regulate critical pathophysiological mechanisms associated with Alzheimer’s disease, encompassing neuroinflammation, synaptic impairment, oxidative stress, metabolic dysregulation, and circadian rhythm disruptions. These neuropeptides offer alternative and frequently under-investigated mechanisms for neuroprotection and disease modulation, in contrast to traditional neurotrophic factors like BDNF or NGF. Preclinical evidence indicates their therapeutic potential; however, clinical application is constrained by issues such as rapid enzymatic degradation, suboptimal bioavailability, restricted blood–brain barrier penetration, and challenges in achieving receptor-specific targeting. To surmount these obstacles, forthcoming research must concentrate on the mechanistic clarification of neuropeptide signaling in disease-relevant scenarios, the creation of stable peptide analogs and chemical modifications to augment half-life and bioactivity, and the adoption of targeted delivery methodologies, including intranasal administration, nanocarrier systems, and controlled release formulations to enhance central nervous system penetration and minimize off-target effects. Furthermore, precision medicine methodologies that utilize patient-specific biomarkers, pharmacogenomics, and genetic profiling may facilitate customized treatments and pinpoint subpopulations most likely to derive benefit. By combining neuropeptide-based therapies with current therapeutic frameworks and fixing current problems with pharmacokinetics and delivery, these molecules have a lot of potential to help make intervention strategies in AD complete and more effective. Ongoing interdisciplinary research that connects molecular pharmacology, bioengineering, and clinical neuroscience will be necessary to turn these promising preclinical results into safe and effective treatments.

## Figures and Tables

**Table 1:** Neuropeptides and their therapeutic potential in AD.

Neuropeptide	Mechanism of Action	Therapeutic Potential in AD	Challenges	Analog Used in Therapeutic Studies
PACAP	Enhances synaptic plasticity and reduces oxidative stress and neuroinflammation	Improves memory and protects neurons from Aβ toxicity	Short half-life and limited BBB penetration	PACAP (6–38), Acetyl-PACAP derivatives
VIP	Exerts anti-inflammatory and neuroprotective effects and regulates neurotransmission	Reduces neuroinflammation and enhances cognitive function	Rapid degradation and poor bioavailability	Aviptadil (RLF-100), Ro 25-1553, PB1046
SP	Modulates neuroinflammation and enhances neuronal survival	Reduces tau phosphorylation and improves synaptic function	Potential for excitotoxicity and dose-dependent effects	Aprepitant analogs, Fosaprepitant derivatives
NPY	Exerts neuroprotective effects and reduces excitotoxicity and the stress response	Enhances neuronal resilience and reduces cognitive decline	Complex receptor interactions and limited BBB permeability	BIBO3304
SST	Inhibits Aβ aggregation and regulates neurotransmission	Restores synaptic balance and prevents cognitive deficits	Levels decline in AD; replacement strategies are needed	Octreotide, Lanreotide, Pasireotide, Vapreotide
CRF	Modulates the stress response and reduces neuroinflammation	Protects against tau hyperphosphorylation and reduces AD-related anxiety	Stress modulation can have dual effects on cognition	Antalarmin, Pexacerfont, CP-154,526

**Table 3:** Therapeutic challenges and opportunities.

Neuropeptide	Therapeutic Potential	Challenges	Current Strategies	References
PACAP	High (neuroprotective)	Short half-life and poor BBB penetration	Intranasal delivery and analogs	[73]
VIP	Moderate	Receptor cross-talk	VPAC2-selective agonists	[176]
SP	Moderate	Pro/anti-inflammatory effects	NK1R antagonists	[177]
PY	High (neuroprotection)	Receptor subtype selectivity	Y2 receptor agonists	[178]
SST	Moderate	Age-related decline and instability	SSTR2-targeted compounds	[179]
CRF	Moderate–High	Stress system complexity	CRF1 receptor antagonists	[180]

## Data Availability

No new data were created or analyzed in this study. Data sharing is not applicable to this article.
